# Supplementary material for: Who Funds Open Data Sharing? Analysis of data availability statements in biomedical publications
Source: bioRxiv. 2026 Jul 20:2026.07.17.739022. Preprint. [Version 1] doi: 10.64898/2026.07.17.739022 (PMC13419376; doi:10.64898/2026.07.17.739022)
Supplement: Supplement 1 [file NIHPP2026.07.17.739022v1-supplement-1.pdf]

bioRxiv preprint doi: https://doi.org/10.64898/2026.07.17.739022; this version posted July 20, 2026. The copyright holder for this preprint (which was not certified by peer review) is the author/funder. This article is a US Government work. It is not subject to copyright under 17 USC 105 and is also made available for use under a CC0 license.

A Supplementary Tables

Table 1: Open data rates among major biomedical research funders. Funders exceeding both the Weibull-derived 5% survival threshold for total funded articles (≥1,708 articles with oddpub v7 coverage) and 50,000 aggregated OpenAlex works, ranked by observed open data rate. Parent funders (e.g., NIH, UKRI) aggregate all child institutes with deduplicated article counts; sub-agency programmes (e.g., NSF directorates, the DOE Office of Science) are folded into their parent agency following the ROR organizational hierarchy, so an article crediting both a programme and its agency is counted once. **% OD (obs.)** is the headline rate: the directly measured open data rate across all articles in each funder's portfolio. *% OD (est.)* is a supplementary modeled estimate that applies journal-level head-to-head PDF vs. XML correction factors to the XML-only portion; it is informative about the magnitude of XML undercount but assumes the head-to-head subset is representative of XML-only articles within each journal. Cell shading: Total Pubs uses a blue-to-red gradient on log scale; both % OD columns share a linear blue-to-red gradient anchored on the observed range. Full rankings for all 1,833 funders are available in the supplementary materials on GitHub.

Funder	Country	Total Pubs	Open Data	% OD (obs.)	*% OD (est.)*
Agence Nationale de la Recherche	France	5,997	1,461	24.4	25.7
Centre National de la Recherche Scientifique	France	1,998	488	24.4	24.9
Austrian Science Fund	Austria	1,918	437	22.8	23.9
Chinese Academy of Sciences	China	3,993	905	22.7	25.4
Swiss National Science Foundation	Switzerland	5,600	1,263	22.6	26.0
Wellcome Trust	UK	8,809	1,948	22.1	24.4
United States Department of Agriculture	USA	4,329	941	21.7	24.5

Continued on next page...

bioRxiv preprint doi: https://doi.org/10.64898/2026.07.17.739022; this version posted July 20, 2026. The copyright holder for this preprint (which was not certified by peer review) is the author/funder. This article is a US Government work. It is not subject to copyright under 17 USC 105 and is also made available for use under a CC0 license.

Funder	Country	Total Pubs	Open Data	% OD (obs.)	*% OD (est.)*
Deutsche Forschungsgemeinschaft	Germany	14,292	3,048	21.3	23.4
National Science Foundation	USA	25,256	5,139	20.3	22.8
Research Foundation Flanders	Belgium	1,897	384	20.2	22.3
Department of Energy	USA	5,788	1,166	20.1	22.2
Netherlands Organisation for Scientific Research	Netherlands	3,439	665	19.3	22.2
Bundesministerium fur Bildung und Forschung	Germany	4,579	868	19.0	21.9
Ministry of Education, Culture, Sports, Science and Technology	Japan	3,868	720	18.6	23.4
American Heart Association	USA	1,909	354	18.5	19.5
Australian Research Council	Australia	1,800	328	18.2	21.1
State Research Agency (Spain)	Spain	4,933	887	18.0	19.8
Natural Sciences and Engineering Research Council of Canada	Canada	5,300	948	17.9	20.5
UK Research and Innovation	UK	21,932	3,853	17.6	21.4
European Commission	EU	28,782	4,970	17.3	20.4
U.S. Department of Defense	USA	6,431	1,091	17.0	19.7
China Scholarship Council	China	3,198	538	16.8	19.8
Swedish Research Council	Sweden	4,900	818	16.7	20.9
National Institutes of Health	USA	85,144	13,978	16.4	18.7
Research Council of Norway	Norway	1,950	315	16.2	20.2
European Regional Development Fund	EU	5,962	948	15.9	18.2
Ministry of Science and Innovation (Spain)	Spain	4,817	759	15.8	18.3
Centers for Disease Control and Prevention	USA	4,082	596	14.6	19.1
Australian Government	Australia	2,397	349	14.6	18.9
Canadian Institutes of Health Research	Canada	6,111	865	14.2	17.7
Science and Technology Commission of Shanghai Municipality	China	2,544	358	14.1	17.7
Fundamental Research Funds for the Central Universities	China	7,174	1,004	14.0	18.6
China Postdoctoral Science Foundation	China	6,130	861	14.0	18.2
National Key Research and Development Program	China	21,352	2,977	13.9	17.9
National Health and Medical Research Council	Australia	4,410	611	13.9	18.4
Russian Science Foundation	Russia	2,157	296	13.7	14.9
Fundacao de Amparo a Pesquisa do Estado de Sao Paulo	Brazil	3,040	414	13.6	17.2
Natural Science Foundation of Guangdong Province	China	2,771	377	13.6	17.9
National Natural Science Foundation of China	China	95,104	12,511	13.2	17.7
U.S. Department of Veterans Affairs	USA	3,279	425	13.0	16.2

Continued on next page...

bioRxiv preprint doi: https://doi.org/10.64898/2026.07.17.739022; this version posted July 20, 2026. The copyright holder for this preprint (which was not certified by peer review) is the author/funder. This article is a US Government work. It is not subject to copyright under 17 USC 105 and is also made available for use under a CC0 license.

Funder	Country	Total Pubs	Open Data	% OD (obs.)	*% OD (est.)*
Fundacao para a Ciencia e a Tecnologia	Portugal	3,348	424	12.7	15.1
Zhejiang Provincial Natural Science Foundation	China	3,349	422	12.6	17.7
Instituto de Salud Carlos III	Spain	3,398	426	12.5	16.1
Natural Science Foundation of Jiangsu Province	China	2,526	315	12.5	16.8
Ministry of Education, India	India	2,067	255	12.3	17.1
Government of Jiangsu Province	China	4,964	591	11.9	16.1
Natural Science Foundation of Shandong Province	China	4,170	492	11.8	16.1
National Council for Scientific and Technological Development (CNPq)	Brazil	6,728	775	11.5	15.9
Japan Society for the Promotion of Science	Japan	20,612	2,360	11.4	16.2
National Research Foundation	South Africa	11,435	1,229	10.7	15.3
National Institute for Health Research	UK	8,924	914	10.2	16.3
Coordenacao de Aperfeicoamento de Pessoal de Nivel Superior	Brazil	6,134	612	10.0	14.7
National Research Foundation of Korea	Korea	10,745	970	9.0	14.0
Pfizer	USA	4,381	352	8.0	14.6
Ministry of Science and ICT, South Korea	Korea	6,574	502	7.6	12.8
Eli Lilly and Company	USA	2,699	190	7.0	14.6

Table 2: Open data rates among top biomedical journals. Journals exceeding the Weibull-derived 5% survival threshold for total articles (≥1,815 articles with oddpub v7 coverage), ranked by observed open data rate. % OD (obs.) shows the directly measured rate; % OD (est.) applies journal-level correction factors from head-to-head PDF vs. XML comparison to estimate the true rate for articles with XML-only coverage. Cell shading: Total Pubs uses a blue-to-red gradient on log scale; % OD columns use a linear blue-to-red gradient. Full rankings for all 1,388 journals are available in the supplementary materials on GitHub.

Journal	Total Pubs	Open Data	% OD (obs.)	% OD (est.)
Scientific Data	2,421	1,588	65.6	65.6
iScience	3,514	1,785	50.8	50.8
Communications Biology	2,503	1,181	47.2	60.1
Nature Communications	15,129	6,835	45.2	55.9
Frontiers in Microbiology	3,850	1,443	37.5	50.2
BMC Plant Biology	1,982	668	33.7	42.5
Frontiers in Plant Science	3,649	764	20.9	30.8
Microorganisms	3,132	610	19.5	25.0
Viruses	2,076	356	17.1	26.7
Frontiers in Veterinary Science	2,238	363	16.2	23.9
Ecology and Evolution	2,325	372	16.0	55.1
Chemical Science	2,486	378	15.2	15.2

Continued on next page...

bioRxiv preprint doi: https://doi.org/10.64898/2026.07.17.739022; this version posted July 20, 2026. The copyright holder for this preprint (which was not certified by peer review) is the author/funder. This article is a US Government work. It is not subject to copyright under 17 USC 105 and is also made available for use under a CC0 license.

Journal	Total Pubs	Open Data	% OD (obs.)	% OD (est.)
Frontiers in Immunology	5,876	859	14.6	19.8
PLoS ONE	24,182	3,427	14.2	23.8
Frontiers in Endocrinology	2,649	361	13.6	16.8
Frontiers in Nutrition	2,356	305	12.9	19.8
Genes	1,815	233	12.8	21.0
Scientific Reports	49,520	5,775	11.7	14.5
BMC Infectious Diseases	1,953	226	11.6	14.8
Poultry Science	1,824	210	11.5	16.0
PeerJ	2,512	272	10.8	16.0
BMC Public Health	5,185	501	9.7	12.1
BMC Cancer	2,277	222	9.7	12.1
Frontiers in Psychology	4,285	379	8.8	12.8
Science Advances	3,594	302	8.4	16.2
Proceedings of the National Academy of Sciences	4,134	320	7.7	16.3
Frontiers in Public Health	5,387	412	7.6	14.4
Frontiers in Pharmacology	3,697	279	7.5	12.8
Heliyon	16,600	1,231	7.4	16.0
International Journal of Molecular Sciences	14,269	1,008	7.1	12.2
Animals	4,759	320	6.7	10.4
Plants	4,653	294	6.3	10.4
Frontiers in Psychiatry	2,334	142	6.1	10.2
Advanced Science	4,305	264	6.1	13.2
BMC Oral Health	2,196	112	5.1	6.6
Frontiers in Medicine	3,462	167	4.8	6.8
RSC Advances	4,574	208	4.5	4.5
Frontiers in Oncology	4,069	183	4.5	6.9
BMC Health Services Research	2,205	88	4.0	5.1
Frontiers in Neurology	2,291	86	3.8	4.8
Molecules	7,085	255	3.6	5.4
Medicine	4,702	140	3.0	16.0
ACS Omega	5,973	153	2.6	16.0
Life	1,867	43	2.3	3.2
Foods	5,262	111	2.1	2.6
BMC Medical Education	2,148	46	2.1	2.7
Cancers	4,121	77	1.9	4.3
Pharmaceuticals	1,846	35	1.9	4.2
Biomedicines	3,002	58	1.9	2.7
Nutrients	4,728	92	1.9	3.7
BMJ Open	4,923	77	1.6	16.0
International Journal of Environmental Research and Public Health	2,018	18	0.9	3.1
Diagnostics	3,400	25	0.7	1.4
Healthcare	3,137	21	0.7	2.5
Medicina	2,321	11	0.5	1.9
Sensors	10,333	39	0.4	1.0
Polymers	4,477	18	0.4	1.3
Journal of Clinical Medicine	8,679	26	0.3	0.9
Nanomaterials	2,402	7	0.3	1.2
JAMA Network Open	2,805	6	0.2	15.9

Continued on next page...

bioRxiv preprint doi: https://doi.org/10.64898/2026.07.17.739022; this version posted July 20, 2026. The copyright holder for this preprint (which was not certified by peer review) is the author/funder. This article is a US Government work. It is not subject to copyright under 17 USC 105 and is also made available for use under a CC0 license.

Journal	Total Pubs	Open Data	% OD (obs.)	% OD (est.)
Materials	8,035	13	0.2	0.6
Cureus	28,751	41	0.1	0.2
Radiology Case Reports	1,875	1	0.1	16.0
International Journal of Surgery Case Reports	2,131	1	0.0	16.0
